# An open-label multicenter study to assess the safety of dextromethorphan/quinidine in patients with pseudobulbar affect associated with a range of underlying neurological conditions

**DOI:** 10.1185/03007995.2014.940040

**Published:** 2014-07-28

**Authors:** Gary L. Pattee, James P. Wymer, Catherine Lomen-Hoerth, Stanley H. Appel, Andrea E. Formella, Laura E. Pope

**Affiliations:** ^a^ Neurology Associates Lincoln, NEUSA; ^b^ The Neurosciences Institute, Albany Medical Center Albany, NYUSA; ^c^ Department of Neurology, University of California San Francisco, CAUSA; ^d^ Department of Neurology, Methodist Neurological Institute, The Methodist Hospital Research Institute, The Methodist Hospital Houston, TXUSA; ^e^ Avanir Pharmaceuticals Inc. Aliso Viejo, CAUSA

**Keywords:** Dextromethorphan/quinidine, Pseudobulbar affect, Safety, Tolerability

## Abstract

**Background:**

Pseudobulbar affect (PBA) is associated with neurological disorders or injury affecting the brain, and characterized by frequent, uncontrollable episodes of crying and/or laughing that are exaggerated or unrelated to the patient’s emotional state. Clinical trials establishing dextromethorphan and quinidine (DM/Q) as PBA treatment were conducted in patients with amyotrophic lateral sclerosis (ALS) or multiple sclerosis (MS). This trial evaluated DM/Q safety in patients with PBA secondary to any neurological condition affecting the brain.

**Objective:**

To evaluate the safety and tolerability of DM/Q during long-term administration to patients with PBA associated with multiple neurological conditions.

**Methods:**

Fifty-two-week open-label study of DM/Q 30/30 mg twice daily. Safety measures included adverse events (AEs), laboratory tests, electrocardiograms (ECGs), vital signs, and physical examinations.

**Clinical trial registration:**

#NCT00056524.

**Results:**

A total of 553 PBA patients with >30 different neurological conditions enrolled; 296 (53.5%) completed. The most frequently reported treatment-related AEs (TRAEs) were nausea (11.8%), dizziness (10.5%), headache (9.9%), somnolence (7.2%), fatigue (7.1%), diarrhea (6.5%), and dry mouth (5.1%). TRAEs were mostly mild/moderate, generally transient, and consistent with previous controlled trials. Serious AEs (SAEs) were reported in 126 patients (22.8%), including 47 deaths, mostly due to ALS progression and respiratory failure. No SAEs were deemed related to DM/Q treatment by investigators. ECG results suggested no clinically meaningful effect of DM/Q on myocardial repolarization. Differences in AEs across neurological disease groups appeared consistent with the known morbidity of the primary neurological conditions. Study interpretation is limited by the small size of some disease groups, the lack of a specific efficacy measure and the use of a DM/Q dose higher than the eventually approved dose.

**Conclusions:**

DM/Q was generally well tolerated over this 52 week trial in patients with PBA associated with a wide range of neurological conditions.

## Introduction

Pseudobulbar affect (PBA) is a neurological condition that exerts a significant health burden on patients and caregivers^1^. PBA is associated with a wide range of neurological disorders and is characterized by frequent, sudden, uncontrollable episodes of crying and/or laughing that are greatly exaggerated or contrary to the patient’s emotional state^2^. Available prevalence studies suggest PBA is present in at least 5% of Parkinson’s disease (PD), 10% of multiple sclerosis (MS), stroke, traumatic brain injury (TBI), and Alzheimer’s disease (AD), 20% of progressive supranuclear palsy, and up to 50% of amyotrophic lateral sclerosis (ALS) patients^3–11^.

PBA is thought to arise from disruption of corticobulbar and cerebellar/pontine pathways controlling emotional expression^12–14^. These pathways may be disrupted by multiple neurological conditions, yet the ensuing clinical manifestations of PBA are indistinguishable, consistent with a common etiology across disorders^12–14^. The combination of dextromethorphan and quinidine (DM/Q) is the first pharmacotherapy approved by the US Food and Drug Administration (FDA) and European Medicines Authority (EMA) for treating PBA^15^,^16^. Dextromethorphan (DM) has many pharmacological actions, including uncompetitive *N*-methyl-D-aspartate (NMDA) receptor antagonism^17^, sigma-1 receptor agonism^18^, and serotonin reuptake inhibition, among others; the precise mechanism(s) accounting for PBA suppression is/are unknown^19^. DM is co-administered with low-dose quinidine, a potent CYP2D6 inhibitor that reduces rapid first-pass metabolism of DM. This inhibition increases DM bioavailability and half-life^20^,^21^. The efficacy, safety, and tolerability of DM/Q as PBA treatment was established in three controlled clinical trials lasting 4^22^ or 12^23^,^24^ weeks, using fixed-dose combinations of twice daily DM/Q 30/30 mg^22^,^23^, 30/10 mg^24^, or 20/10 mg^24^ in patients with ALS^22^,^24^ or MS^23^,^24^. The present trial was designed to provide long-term safety data using the higher DM/Q 30/30 mg dose in patients with PBA, regardless of primary neurological condition.

## Methods

### Study design

Study 02-AVR-107 (NCT00056524) is a multicenter, 52 week, open-label safety trial. The trial began in March 2003 at 44 sites internationally (39 in the US, four in Israel, and one in Serbia and Montenegro). An extension was added to allow completing patients the option to remain on treatment past 1 year. The trial was terminated by the sponsor in June 2007, following the decision to pursue development of a lower DM/Q dose. Patients were instructed to take DM/Q 30/30 mg in the evening for 7 days and then twice daily thereafter. Patients kept a diary of dosage times and recorded any adverse events (AEs). Clinic visits occurred at screening, baseline (Day 1), and after 1, 4, 8, and 12 months of treatment or at patient discontinuation. During months when no clinic visit was scheduled, patients were contacted by telephone and asked about medication compliance and AEs.

### Study population

Eligible patients were 18 to 75 years of age with a clinical diagnosis of PBA. For the purposes of this study, PBA was defined as ‘a syndrome characterized by outbursts of crying and/or laughing that occur spontaneously and inappropriately given the context in which they occur’. No specific threshold for severity of PBA symptoms was required for study entry. Patients were required to have an electrocardiogram (ECG) with no evidence of rate-corrected QT interval (QTc) prolongation (≥450 msec in men; ≥470 msec in women), heart block (isolated right bundle branch block without clinical history of heart disease was allowable), sinus bradycardia (<50 bpm), ventricular tachycardia, multifocal ventricular ectopic beats (any frequency), or unifocal ventricular ectopic beats (>5/min). Patients with ALS were required to have a vital capacity ≥50% at baseline. Patients completing prior controlled studies of twice daily DM/Q 30/30 mg to treat PBA in MS^23^ or ALS^22^ were also eligible to participate, provided they met all eligibility requirements at the time of enrollment in this study.

Exclusion criteria were myasthenia gravis; a history of ventricular tachycardia or torsades de pointes; sensitivity to quinidine or opiate drugs; major psychiatric disturbance; a history of substance abuse in the past 2 years; any major systemic disease that would interfere with interpretation of study results (e.g., malignancy, uncontrolled diabetes, dilated cardiac myopathy, ischemic or valvular heart disease); hypotension (systolic blood pressure [BP] <100 mmHg); a history of postural or any unexplained syncope; renal, hepatic, or pulmonary disease; or clinically significant deviations in standard laboratory tests. Female patients could not be pregnant or breastfeeding; those with child-bearing potential were required to use an established method of birth control.

Patients were allowed to continue existing medications, except for the following starting from 1 week before DM/Q initiation: ketoconazole, voriconazole, verapamil, diltiazem, nifedipine, sodium bicarbonate, carbonic anhydrase inhibitors, thiazide diuretics (unless urine pH ≤6.5 and on thiazide for ≥1 month at enrollment), warfarin, haloperidol, monoamine oxidase inhibitors, and tricyclic antidepressants at doses >75 mg/day (>20 mg/day for desipramine; >15 mg/day for protriptyline). Other than the study drug, medications containing dextromethorphan or quinidine were prohibited.

### Safety/tolerability assessments

Safety and tolerability measures included: AEs recorded in patient diaries and during clinic visits and telephone contacts (serious adverse events [SAEs], including deaths, were required to be reported through the 30 days following final dose); vital signs (all visits); resting 12-lead ECG (screening, Day 29, and Final Visit at Week 52 or discontinuation); clinical laboratory values, including serum chemistry, hematology, and urinalysis (screening, Day 29, and Final Visit); and physical examination (screening and Final Visit).

### Pharmacokinetic assessments

A blood sample was obtained on Day 29 within 12 h after dosing to determine plasma concentrations of DM, quinidine, and the DM metabolite dextrorphan (DX).

### Safety-data analyses

The safety population comprised all patients receiving at least one DM/Q dose. The numbers of patients experiencing AEs were summarized by body system, relationship to study drug, and severity. SAEs and AEs that resulted in study discontinuation were also summarized descriptively. Additionally, patient demographics, disposition, concomitant medications, and AEs were analyzed by primary neurological condition. Likelihood-ratio chi-square tests were used to compare AE incidence among disease groups. Assignment of patients to subgroups for analysis by primary neurological condition was made based on similarities among nosological characteristics and without knowledge of patient data or safety outcome.

Clinical laboratory values were summarized descriptively; shifts between normal, low, or high values were analyzed using McNemar’s test. Changes in physical-examination findings (normal vs. abnormal) were assessed by McNemar’s test. Summary statistics of absolute values and percentage change from screening value were provided for systolic and diastolic BP, heart rate, respiratory rate, QT interval, QTc interval, ventricular rate, PR interval, and QRS duration. Clinically significant abnormalities were documented.

### Ethics and Good Clinical Practice

The study was conducted in conformity with the standards of Good Clinical Practices and the Declaration of Helsinki. Eligible patients were informed of the study’s purposes and of anticipated AEs that recipients might experience, and ethics committee approval was obtained for each study site. Before any study procedures, a signed informed consent document was obtained.

## Results

A total of 553 patients with PBA were enrolled (494 in the US, 48 in Israel, and 11 in Serbia and Montenegro). Eighty-nine of these patients entered directly from a 12 week, placebo-controlled DM/Q study to treat PBA in MS, and 56 others (9 with ALS and 47 with MS) had been previously exposed to DM and/or quinidine in clinical studies^22^,^23^. Over 30 neurological conditions were represented among the participating cohort. In addition to ALS (*n* = 176) and MS (*n* = 223), 154 participants had PBA secondary to diverse neurological conditions (Table 1). These conditions were categorized into seven disease groups as follows: ALS/motor neuron diseases (MND) (*n* = 199), MS (*n = *223), stroke/cerebrovascular disorders (CBVD) (*n = *51), TBI (*n = *23), PD/movement disorders (*n = *23), AD/dementia (*n = *17), and ‘other PBA’ (*n = *17), as shown in Table 1.

**Table 1.** Categorization of patients’ primary neurological conditions into disease groups (*n*).

AD/dementia (*n = *17)		MS (*n = *223)		Stroke/CBVD (*n = *51)		ALS/MND (*n = *199)		TBI (*n = *23)		PD/movement disorders (*n = *23)		Other PBA (*n = *17)	
AD	15	MS	223	Stroke	46	ALS	176	TBI	21	PD	11	Cerebellar ataxia	3
Frontal lobe dementia	2			Venous angioma	1	Primary lateral sclerosis	16	Head trauma	2	Parkinson syndrome	1	Spinocerebellar ataxia	2
				Pontine AVM	1	Progressive bulbar palsy	5			Atypical PD	1	Cerebral palsy	2
				Post-cerebral aneurysm surgery	1	Bulbar motor neuron disease or degeneration	1			Parkinsonian syndrome	1	Chronic cough	1
				Brain aneurysm	1	Bulbar palsy	1			Progressive supranuclear palsy	5	Small-fiber neuropathy	1
				Subarachnoid hemorrhage	1					Huntington’s disease	1	Leuko-encephalopathy	1
										Choreiform disorder	1	Post-herpes simplex virus	1
										Movement disorder	1	Subdural hematoma	1
										Corticobasilar degeneration	1	Viral meningio-encephalitis	1
												Hydrocephalus	1
												Post-encephalitis	1
												Foster-Kennedy syndrome	1
												None known	1

AD: Alzheimer’s disease; ALS: amyotrophic lateral sclerosis; AVM: arteriovenous malformation; CBVD: cerebrovascular disorders; MND: motor neuron disease; MS: multiple sclerosis; PBA: pseudobulbar affect; PD: Parkinson’s disease; TBI: traumatic brain injury.

### Patient disposition

**Figure 1.**
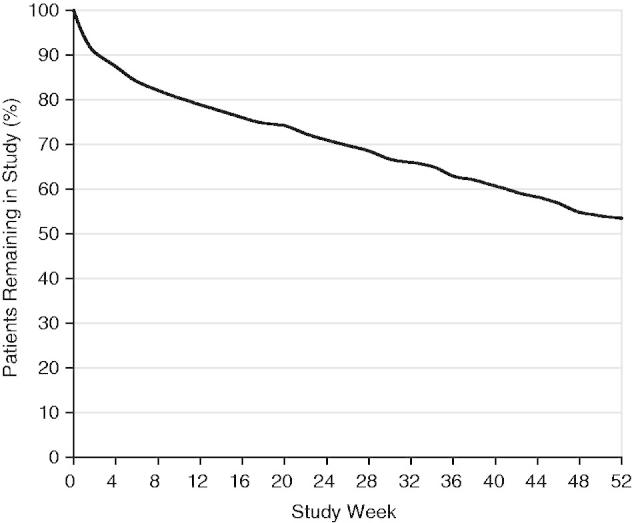
Proportion of patients remaining in study, by study week.

Patient disposition is shown in Table 2 and duration of study participation is shown in Figure 1. Of the 553 patients treated with DM/Q, 382 patients (69.1%) completed at least 6 months of treatment, 300 (54.2%) completed 1 year, and 296 (53.5%) completed the protocol-specified visits. One hundred forty-eight (26.8%) patients dropped out of the trial for either an AE or medication refusal due to an AE; 109 (19.7%) dropped out for other reasons. Of the completers, 262 (88.5%) elected to continue DM/Q in the optional extension. At trial termination, 20 patients (3.6%) remained in the treatment phase and 168 (64.1% of those entering the extension) remained in the extension. This report provides results from the 52 week treatment phase.

**Table 2.** Patient disposition by primary neurological condition^a^.

Patient disposition, *n*	AD/dementia	MS	Stroke/CBVD	TBI	ALS/MND	PD/movement disorders	Other PBA	Total
Enrolled	*n = *17	*n = *223	*n = *51	*n = *23	*n = *199	*n = *23	*n = *17	*N = *553
Treated ≥6 months	10	176	30	15	126	12	13	382
Treated ≥12 months^a^	5	149	27	10	86	11	12	300
Completed treatment phase	4	149	27	9	85	10	12	296
Remaining at study termination	2	8	4	2	4	0	0	20
Did not complete due to:	11	66	20	12	110	13	5	257
AE	4	27	7	1	62	5	1	107
Medication refusal due to AE	2	10	4	2	18	4	1	41
Medication refusal for other than AE	0	7	1	2	3	0	1	14
Withdrew consent	3	15	3	3	9	1	0	34
Lost to follow-up	0	4	4	2	7	0	1	18
Protocol violation	0	2	0	0	1	0	0	3
Other	4	9	5	4	14	3	1	40
Entered extension phase	4	132	22	8	77	8	11	262

AD: Alzheimer’s disease; AE: adverse event; ALS: amyotrophic lateral sclerosis; CBVD: cerebrovascular disorders; MND: motor neuron disease; MS: multiple sclerosis; PBA: pseudobulbar affect; PD: Parkinson’s disease; TBI: traumatic brain injury. ^a^Includes all patients who completed the treatment phase or received at least 365 days of dosing.

### Patient demographics

Patient demographics by primary disease are provided in Table 3. Patients reported a median of 7.0 (range 0–420; mean 16.7 ± 32.6) PBA episodes per week at baseline. Some patients transitioned directly from a previous DM/Q study and therefore may have had low or no PBA episodes at baseline. The median patient age was 52.0 years (range 18–86), 15.9% were ≥65 years old, 58.2% were female, and 90.8% were Caucasian.

**Table 3.** Patient demographics by primary neurological condition.

Category	AD/dementia (*n = *17)	MS (*n = *223)	Stroke/CBVD (*n = *51)	TBI (*n = *23)	ALS/MND (*n = *199)	PD/movement disorders (*n = *23)	Other PBA (*n = *17)
Age (years)							
Mean	63.5	45.7	54.8	45.6	55.6	64.9	49.1
SD	11.20	10.23	10.93	13.81	11.06	8.00	13.71
Median	66.0	47.0	56.0	44.0	57.0	66.0	52.0
Min, max	38, 79	18, 71	30, 80	18, 74	18, 80	50, 86	22, 67
Gender, *n* (%)							
Male	10 (58.8)	49 (22.0)	24 (47.1)	10 (43.5)	116 (58.3)	15 (65.2)	7 (41.2)
Female	7 (41.2)	174 (78.0)	27 (52.9)	13 (56.5)	83 (41.7)	8 (34.8)	10 (58.8)
Ethnicity, *n* (%)							
Caucasian	16 (94.1)	208 (93.3)	43 (84.3)	18 (78.3)	179 (89.9)	22 (95.7)	16 (94.1)
Black	1 (5.9)	5 (2.2)	5 (9.8)	1 (4.3)	5 (2.5)	0 (0)	0 (0)
Asian	0 (0)	1 (0.4)	1 (2.0)	0 (0)	1 (0.5)	0 (0)	0 (0)
Hispanic	0 (0)	8 (3.6)	2 (3.9)	3 (13.0)	9 (4.5)	1 (4.3)	1 (5.9)
Other	0 (0)	1 (0.4)	0 (0)	1 (4.3)	5 (2.5)	0 (0)	0 (0)

AD: Alzheimer’s disease; ALS: amyotrophic lateral sclerosis; CBVD: cerebrovascular disorders; MND: motor neuron disease; MS: multiple sclerosis; PBA: pseudobulbar affect; PD: Parkinson’s disease; SD: standard deviation; TBI: traumatic brain injury.

### Concurrent medications

Patients were taking a median of seven additional medications at baseline (range 0–30) including those for their primary neurological condition and other conditions. The number of baseline medications was similar for study completers and those who discontinued for AEs.

The most frequently reported concurrent chronic medications (taken ≥3 months during treatment) were nonsteroidal anti-inflammatory drugs and other analgesics, antidepressants, lipid-modifying agents, antithrombotics, inhibitors of gastric acid production, vitamins, anticonvulsants, and benzodiazepines (Table 4). Antipsychotics were used chronically by 2.2% of patients.

**Table 4.** Common concurrent medications^a^ by primary neurological condition.

Description, %	AD/dementia (*n = *17)	MS (*n = *223)	Stroke/CBVD (*n = *51)	TBI (*n = *23)	ALS/MND (*n = *199)	PD/movement disorders (*n = *23)	Other PBA (*n = *17)	Total (*N = *553)
NSAIDs	23.5	36.3	19.6	30.4	40.2	17.4	5.9	33.8
Antidepressants	23.5	23.3	31.4	30.4	29.1	34.8	23.5	26.9
Tricyclic antidepressants	0	4.9	2.0	13.0	13.1	4.3	0	7.6
SSRIs	5.9	15.2	21.6	17.4	10.6	13.0	5.9	13.6
Other^b^	17.6	8.1	7.8	8.7	10.1	17.4	17.6	9.8
Lipid modifiers	35.3	22.4	35.3	21.7	20.6	13.0	23.5	23.0
Antithrombotics	35.3	12.1	52.9	17.4	24.6	21.7	5.9	21.5
Analgesics (e.g., acetaminophen)	35.3	25.6	19.6	21.7	14.1	13.0	5.9	19.9
Drugs to inhibit gastric acid production	23.5	15.7	23.5	21.7	22.6	17.4	23.5	19.7
Vitamin E	11.8	7.6	3.9	4.3	37.7	8.7	29.4	18.8
Antiepileptics	17.6	23.8	25.5	43.5	9.0	8.7	29.4	18.8
Multivitamins, plain	5.9	22.4	7.8	8.7	20.1	0	11.8	17.9
Anxiolytics	23.5	16.6	11.8	13.0	20.1	21.7	11.8	17.5
Vitamin C	11.8	12.6	0	4.3	30.7	8.7	0	17.0
Antihistamines for systemic use	17.6	14.8	7.8	26.1	18.1	4.3	17.6	15.6
Vitamin B12 and folic acid	11.8	9.9	7.8	17.4	20.1	4.3	11.8	13.6
Calcium	5.9	20.2	7.8	8.7	7.5	17.4	5.9	13.0
Hypnotics and sedatives	17.6	8.5	3.9	4.3	20.6	17.4	0	12.7
Opioids	11.8	11.7	5.9	8.7	15.1	4.3	11.8	11.9
Laxatives	11.8	9.4	5.9	4.3	17.1	0	11.8	11.4
Misc. herbal	0	13.0	2.0	0	15.1	0	0	10.8
Beta-blocking agents	11.8	6.7	13.7	4.3	13.1	17.4	5.9	10.1
Thyroid preparations	17.6	9.4	9.8	17.4	7.5	13.0	5.9	9.4
ACE inhibitors, plain	17.6	5.8	19.6	13.0	9.0	4.3	11.8	9.0

ACE: angiotensin-converting enzyme; AD: Alzheimer’s disease; ALS: amyotrophic lateral sclerosis; CBVD: cerebrovascular disorders; MND: motor neuron disease; MS: multiple sclerosis; NSAIDs: nonsteroidal anti-inflammatory drugs; PBA: pseudobulbar affect; PD: Parkinson’s disease; SSRI: selective serotonin reuptake inhibitor; TBI: traumatic brain injury. ^a^Used for ≥3 months and taken during the study; drug use represents minimum use in each category and may not include some combination products such as antihistamine cold products, combinations of vitamins and herbals, etc. ^b^Other includes: venlafaxine, 3.6%; bupropion, 3.3%; trazodone, 2.5%; mirtazepine, 1.3%; duloxetine, mianserin, nefazodone, reboxetine, and hydroxytryptophan, 0.2% (1 patient) each.

Common disease-specific therapies included: interferon beta, glatiramer, baclofen, and stimulants in patients with MS; donepezil, and memantine in patients with AD; riluzole in patients with ALS; dopaminergic drugs in patients with PD; antithrombotics and cardiovascular medications in patients with stroke; and antiepileptics in patients with TBI (Table 5).

**Table 5.** Common concurrent disease-specific medications^a^ by primary neurological condition.

AD/dementia (*n = *17)	%
Anticholinesterase (e.g., donepezil)	47.1
Memantine	29.4
MS (*n = *223)	**%**
Interferon beta	45.3
Glatiramer	27.4
Centrally acting muscle relaxants (e.g., baclofen, tizanidine, orphenadrine)	33.2
Urinary antispasmodics (e.g., oxybutynin, tolterodine)	25.6
Psychostimulants (e.g., amphetamine derivatives, modafinil, pemoline)	18.8
Glucocorticoids	5.8
Immunosuppressants (e.g., methotrexate, mycophenolate, azathioprine)	5.8
Mitoxantrone	4.0
4-Aminopyridine	2.2
Stroke/CBVD (*n = *51)	**%**
Aspirin	43.1
Clopidogrel	19.6
Calcium channel blockers (e.g., dihydropyridine)	11.8
TBI (*n = *23)	**%**
Topiramate	13.0
Carbamazepine/oxcarbazepine	13.0
Thyroid preparations	17.4
Psychostimulants (e.g., amphetamine derivatives, modafinil, pemoline)	13.0
Antimigraine (e.g., triptans, selective 5HT1 agonists)	13.0
Glucocorticoids	8.7
ALS/MND (*n = *199)	**%**
Riluzole	47.7
Centrally acting muscle relaxants (e.g., baclofen, tizanidine, orphenadrine)	31.7
Coenzyme Q10	31.2
Tetracycline antibiotics (e.g., minocycline, doxycycline)	17.6
Creatine	15.6
Beta-2-adrenergic agonists, inhalants	14.6
Beta carotene/vitamin A	13.1
Urinary antispasmodics (e.g., oxybutynin, tolterodine)	11.1
Quinine	10.6
Expectorants and mucolytics (e.g., guaifenesin, *n*-acetylcysteine)	9.0
Belladonna alkaloids (e.g., atropine, hyocyamine, scopolamine)	9.0
Glycopyrrolate	7.5
PD/movement disorders (*n = *23)	**%**
Dopamine derivatives (± carbidopa, benserazide, or entacapone)	65.2
Dopamine agonists	30.4
Amantadine	17.4
Selegiline	8.7
Trihexyphenidyl	0.5
Other PBA (*n = *17)	**%**
Phenytoin	11.8
Tiagabine	11.8

5HT1: type 1 5-hydroxytryptamine (serotonin) receptor; AD: Alzheimer’s disease; ALS: amyotrophic lateral sclerosis; CBVD: cerebrovascular disorders; MND: motor neuron disease; MS: multiple sclerosis; PBA: pseudobulbar affect; PD: Parkinson’s disease; TBI: traumatic brain injury. ^a^Used for ≥3 months and taken during the study.

### Adverse events

Over 90% of patients (*n = *508; 91.8%) reported at least one AE during the 52 week trial. The most frequently reported AEs (incidence ≥15%) were nausea, headache, dizziness, fall, and diarrhea. Most AEs were mild to moderate in severity (64.8% of patients with AEs). Table 6 shows the incidence of commonly reported AEs across disease groups.

**Table 6.** Incidence of adverse events reported by ≥5% and at least two patients in any primary-neurological-condition category (safety population).

Preferred term, % (*n*)	AD/dementia (*n = *17)	MS (*n = *223)	Stroke/ CBVD (*n = *51)	TBI (*n = *23)	ALS/MND (*n = *199)	PD/movement disorders (*n = *23)	Other PBA (*n = *17)	*p* Value^a^	Total (*N = *553)
Any AE	70.6 (12)	89.7 (200)	90.2 (46)	82.6 (19)	98.0 (195)	91.3 (21)	88.2 (15)	0.0004	91.9 (508)
Nausea	11.8 (2)	23.8 (53)	19.6 (10)	13.0 (3)	31.7 (63)	8.7 (2)	23.5 (4)	0.0544	24.8 (137)
Headache	5.9 (1)	25.1 (56)	15.7 (8)	26.1 (6)	22.1 (44)	26.1 (6)	29.4 (5)	0.4613	22.8 (126)
Dizziness	5.9 (1)	21.5 (48)	19.6 (10)	21.7 (5)	17.1 (34)	30.4 (7)	17.6 (3)	0.5201	19.5 (108)
Fall	17.6 (3)	13.5 (30)	7.8 (4)	13.0 (3)	22.6 (45)	21.7 (5)	5.9 (1)	0.0674	16.5 (91)
Diarrhea	23.5 (4)	15.7 (35)	11.8 (6)	13.0 (3)	19.1 (38)	0 (0)	23.5 (4)	0.2426	16.3 (90)
Fatigue	17.6 (3)	16.6 (37)	7.8 (4)	39.1 (9)	11.1 (22)	13.0 (3)	17.6 (3)	0.0141	14.6 (81)
Weakness	0 (0)	14.8 (33)	3.9 (2)	4.3 (1)	18.6 (37)	4.3 (1)	11.8 (2)	0.0245	13.7 (76)
Nasopharyngitis	0 (0)	16.1 (36)	9.8 (5)	13.0 (3)	9.5 (19)	4.3 (1)	11.8 (2)	0.1944	11.9 (66)
Somnolence	5.9 (1)	4.9 (11)	15.7 (8)	4.3 (1)	15.6 (31)	26.1 (6)	11.8 (2)	0.0019	10.8 (60)
Arthralgia	0 (0)	14.3 (32)	5.9 (3)	0 (0)	10.1 (20)	13.0 (3)	5.9 (1)	0.1334	10.7 (59)
Cough	5.9 (1)	10.3 (23)	3.9 (2)	13.0 (3)	14.6 (29)	4.3 (1)	0 (0)	0.1633	10.7 (59)
Ecchymosis	5.9 (1)	8.1 (18)	5.9 (3)	4.3 (1)	12.6 (25)	13.0 (3)	17.6 (3)	0.4343	9.8 (54)
Pain in limb	0 (0)	13.0 (29)	11.8 (6)	0 (0)	8.5 (17)	0 (0)	5.9 (1)	0.1056	9.6 (53)
Constipation	11.8 (2)	5.4 (12)	5.9 (3)	0 (0)	15.1 (30)	4.3 (1)	11.8 (2)	0.0124	9.0 (50)
Insomnia	0 (0)	6.7 (15)	7.8 (4)	8.7 (2)	13.6 (27)	0 (0)	11.8 (2)	0.1004	9.0 (50)
Back pain	5.9 (1)	9.9 (22)	5.9 (3)	0 (0)	9.0 (18)	4.3 (1)	5.9 (1)	0.6684	8.3 (46)
Dysphagia	0 (0)	1.8 (4)	3.9 (2)	0 (0)	20.1 (40)	0 (0)	0 (0)	<0.0001	8.3 (46)
Dry mouth	5.9 (1)	5.8 (13)	9.8 (5)	0 (0)	8.5 (17)	13.0 (3)	0 (0)	0.4119	7.1 (39)
Edema lower limb	5.9 (1)	3.6 (8)	7.8 (4)	8.7 (2)	9.5 (19)	8.7 (2)	11.8 (2)	0.3244	6.9 (38)
Respiratory failure	0 (0)	0 (0)	0 (0)	0 (0)	19.1 (38)	0 (0)	0 (0)	<0.0001	6.9 (38)
Sore throat	5.9 (1)	9.0 (20)	3.9 (2)	4.3 (1)	7.0 (14)			0.4887	6.9 (38)
Urinary tract infection	0 (0)	9.9 (22)	5.9 (3)	4.3 (1)	4.5 (9)	4.3 (1)	11.8 (2)	0.2970	6.9 (38)
Dyspnea	0 (0)	3.6 (8)	3.9 (2)	8.7 (2)	11.6 (23)	4.3 (1)	5.9 (1)	0.0399	6.7 (37)
Fatigue aggravated	0 (0)	9.4 (21)	3.9 (2)	0 (0)	5.0 (10)	0 (0)	5.9 (1)	0.1663	6.1 (34)
Laceration	0 (0)	5.8 (13)	5.9 (3)	0 (0)	8.0 (16)	4.3 (1)	5.9 (1)	0.6724	6.1 (34)
MS aggravated	0 (0)	14.3 (32)	0 (0)	0 (0)	0 (0)	0 (0)	0 (0)	<0.0001	5.8 (32)
Pyrexia	5.9 (1)	7.6 (17)	3.9 (2)		5.0 (10)	4.3 (1)	5.9 (1)	0.7549	5.8 (32)
Vomiting	5.9 (1)	7.6 (17)	2.0 (1)	8.7 (2)	5.0 (10)	4.3 (1)	0 (0)	0.6161	5.8 (32)
Muscle spasms	0 (0)	6.7 (15)	0 (0)	8.7 (2)	6.0 (12)	0 (0)	5.9 (1)	0.3610	5.4 (30)
Nasal congestion	0 (0)	6.3 (14)	3.9 (2)	4.3 (1)	6.0 (12)	0 (0)	0 (0)	0.6634	5.2 (29)
Anxiety	0 (0)	3.6 (8)	0 (0)	4.3 (1)	7.5 (15)	13.0	5.9 (1)	0.1138	5.1 (28)
Joint stiffness	0 (0)	5.4 (12)	2.0 (1)	0 (0)	6.5 (13)	0 (0)	11.8 (2)	0.3292	5.1 (28)

AD: Alzheimer’s disease; AE: adverse event; ALS: amyotrophic lateral sclerosis; CBVD: cerebrovascular disorders; MND: motor neuron disease; MS: multiple sclerosis; PBA: pseudobulbar affect; PD: Parkinson’s disease; TBI: traumatic brain injury. ^a^Chi-square test.

Although chi-square tests were performed for each AE, resulting *p* values must be regarded as exploratory due to multiple comparisons and the small size of some groups. Differences in AE incidence across disease groups appeared consistent with the expected manifestations of the primary neurological conditions. For example, respiratory failure occurred exclusively, and dysphagia and dyspnea predominantly, in ALS/MND patients; fatigue was more than twice as common in TBI patients; somnolence was most common in PD/movement disorders patients; weakness was most common in ALS/MND and MS patients; and constipation was most common in AD/dementia, ALS/MND, and ‘other PBA’ patients.

Because of the high morbidity associated with several neurological conditions, it seems particularly important to also note the frequency of AEs that investigators considered possibly related to DM/Q treatment (TRAEs) (Table 7). Most TRAEs (91%) were mild-to-moderate in severity. Only seven occurred with incidence ≥5%: nausea (11.8%), dizziness (10.5%), headache (9.9%), somnolence (7.2%), fatigue (7.1%), diarrhea (6.5%), and dry mouth (5.1%).

**Table 7.** Incidence of treatment-related^a^ adverse events by primary neurological condition (safety population).

Preferred term, %	AD/dementia (*n = *17)	MS (*n = *223)	Stroke/CBVD (*n = *51)	TBI (*n = *23)	ALS/MND (*n = *199)	PD/movement disorders (*n = *23)	Other PBA (*n = *17)	Total (*N = *553)
Any TRAE	23.5	39.9	51.0	47.8	59.3	60.9	52.9	49.0
Nausea	0	8.1	7.8	4.3	19.1	4.3	17.6	11.8
Dizziness (excluding vertigo)	0	10.3	7.8	13.0	10.1	21.7	17.6	10.5
Headache NOS	0	9.4	5.9	8.7	11.6	21.7	5.9	9.9
Somnolence	0	2.7	5.9	0	13.1	13.0	11.8	7.2
Fatigue	5.9	5.8	2.0	26.1	7.5	4.3	11.8	7.1
Diarrhea NOS	11.8	4.0	3.9	4.3	9.0	0	23.5	6.5
Dry mouth	0	3.1	5.9	0	8.0	8.7	0	5.1

Additional TRAEs that occurred in at least 5% of patients and in two or more patients in any disease category were stroke/CBVD: insomnia (not elsewhere classified), *n = *3 (5.9%); TBI: lethargy, *n = *3 (13.0%), abnormal dreams, *n = *2 (8.7%); ALS/MND: constipation, *n = *11 (5.5%); PD/movement disorders: confusion, *n = *2 (8.7%); other PBA: joint stiffness, *n = *2 (11.8%); flatulence, *n = *2 (11.8%); dyspepsia, *n = *2 (11.8%). AD: Alzheimer’s disease; ALS: amyotrophic lateral sclerosis; CBVD: cerebrovascular disorders; MND: motor neuron disease; MS: multiple sclerosis; NOS: not otherwise specified; PBA: pseudobulbar affect; PD: Parkinson’s disease; TRAE: treatment-related adverse event. ^a^TRAEs that occurred in at least 5% of total patients; treatment-related categories included possible, probable, highly probable, and missing.

To further assess the time course of these seven TRAEs, a post hoc analysis was conducted evaluating time to onset, duration, recurrence, and percentage of total study days with TRAE present (persistence) (Online Resource 1). Common TRAEs generally occurred early in therapy and were transient; both median time to onset and duration ranged from 4 to 8 days for all but fatigue (median duration 16 days). TRAEs did not recur in most patients; the highest incidence of recurrence was headache (42% recurrence after initial episode). TRAE presence as a percentage of total treatment days was low, ranging from a median of 2% for dizziness and headache to 22% for fatigue and sleepiness. Inclusion of patients who discontinued prior to AE resolution can reduce estimates of AE duration while increasing estimates of percentage of treatment days with AE present (by decreasing total treatment days). Omitting these patients from this analysis did not appreciably affect median duration but did decrease estimates of AE persistence (Online Resource 1).

### Serious adverse events

The overall incidence of SAEs was 22.8%; no SAE was reported to be related to study medication. Most SAEs occurred in patients with ALS/MND, including all SAEs of respiratory failure, dysphagia, and pneumonia (Online Resource 2). Except for respiratory events in ALS and disease exacerbation in MS, there was no evidence of increased frequency of SAEs specific to any neurological disorder.

Forty-seven deaths occurred during the 52 week trial, most attributed to ALS progression. Thirty-one of the 39 ALS deaths were attributed to ALS-related respiratory failure or similar respiratory events. The remaining eight were attributed to ‘ALS progression’ (*n = *3), cardiac arrest (*n = *2), pneumonia (*n = *1), infection (*n = *1), and cardiorespiratory arrest and epistaxis (*n = *1). One additional ALS/MND group patient with primary lateral sclerosis died of ‘cause unknown’. Following study completion, an investigator query about all ALS patient deaths provided an estimated median time from ALS diagnosis to death of 29 months. (Actual date of death was obtained for 117 patients; the remainder were censored at date of last contact.) The three deaths in MS patients were attributed to acute myelomonocytic leukemia, myocardial infarction (patient with resolving flu-like illness and extensive pre-existing skin breakdown; received DM/Q for 8 days and then discontinued for recurrent flu-like illness, hallucinations, and lethargy; and died 5 days later), and myocardial infarction and sepsis (patient with pulmonary embolism and sepsis). Of the remaining four deaths, two were attributed to stroke (patient with AD and prior stroke history who died on Day 217, and a patient with prior stroke history who died on Day 41); one was attributed to cardiac arrest (patient with AD and history of acute coronary syndrome and hypertension); and one patient committed suicide on Day 104 (patient with spinocerebellar ataxia and reactive depression). In all cases, death was considered unlikely related to DM/Q treatment. All deaths occurring during the complete DM/Q clinical development program were additionally reviewed by a group of consulting cardiologists and neurologists as part of the FDA review process for the DM/Q application for treatment of PBA. Their findings corroborated the investigators’ initial assessment that the deaths were not related to treatment but to progression of underlying neurological or other medical conditions.

### AEs leading to discontinuation

One hundred forty-nine patients (26.9%) withdrew from the trial or refused further study medication due to AEs (Online Resource 3). This number includes one discontinuation during the extension for an AE beginning in the treatment phase. AE dropout was more frequent in ALS/MND (*n = *80; 40.2% of ALS/MND patients), PD/movement disorders (*n = *9; 39.1% of PD/movement disorders patients), and AD/dementia (*n = *6; 35.3% of AD/dementia patients), and less frequent in stroke/CBVD (*n = *11; 21.5%), MS (*n = *37; 16.6%), TBI (*n = *3; 13.0%), and ‘other PBA’ (*n = *2; 11.8%). The most common AEs leading to discontinuation were respiratory failure in ALS/MND patients (14.1% of ALS/MND group; 5.1% of total), nausea (3.3%), dizziness (2.9%), headache (2.5%), and diarrhea (1.8%). Other than respiratory failure and nausea in the ALS/MND group and dizziness and headache in the PD/movement disorders group, discontinuation due to AEs appeared similar regardless of primary neurological disorder. Approximately half of the AE-related dropouts, (*n = *74 or 13.5% of total patients) included AEs that were judged to be at least possibly related to treatment. In these patients, the median time to discontinuation was 16.5 days (mean 31.4; range 1–243).

### Clinical laboratory values and vital signs

Laboratory test results remained stable during the trial. Shift tables showed no changes of clinical relevance. There were no clinically relevant changes in mean systolic and diastolic BP, heart rate, respiratory rate, or body temperature at any visit. Increased BP was recorded as an AE in 11 patients (2.0%), and increased systolic BP was recorded as an AE in one patient; however, these were considered ‘not related’ or ‘unlikely related’ to DM/Q treatment in all but one patient (‘possibly related’).

### Physical examinations

No abnormal physical findings related to study-drug treatment were observed.

### Electrocardiography

Mean ECG values showed no clinically relevant post-baseline changes (Online Resource 4). QTc intervals showed a small mean increase (QTcF 3.2; QTcB 4.6 msec) from baseline to final measurement. Five patients had QTc increases ≥60 msec; however, all values remained <500 msec during the 52 week treatment phase.

### Pharmacokinetics

Blood samples were provided by 202 patients on Day 29. Mean (standard deviation) plasma concentrations were DM: 92.7 (45.6) ng/mL, DX: 78.0 (43.4) ng/mL, and quinidine: 0.15 (0.09) μg/mL. Mean DM concentration was similar to that reported in previous placebo-controlled trials using this dosage^22^,^23^.

## Discussion

Long-term administration of DM/Q was well tolerated in this large, open-label study of patients with PBA associated with a wide range of neurological conditions. These results provide important safety data for DM/Q, the only approved medication for PBA, a common and distressing disorder experienced by patients with neurologic disease or injury. The low incidence of treatment-emergent AEs with DM/Q used at a higher dose than the approved dose, and occurring in a diverse, clinic-based, ‘real-world’ population, provides reassuring clarity for physicians on the risks of this treatment. The overall incidence of reported AEs is consistent with the trial length and chronic and often progressive nature of the underlying neurological conditions. Although the majority (90%) of patients reported at least one AE, only 49% reported AEs possibly related to DM/Q treatment, and only 13.4% discontinued for TRAEs. The most frequently reported AEs were consistent with those reported at higher incidence than controls in shorter, double-blind DM/Q 30/30 mg trials in PBA patients with ALS or MS^22^,^23^. In a 4 week study in ALS patients^22^, the most commonly reported AEs (incidence ≥15% and more than controls) among DM/Q recipients (*n = *70) were nausea, dizziness, fatigue, diarrhea, and headache. In a 12 week study in MS patients^23^, the most commonly reported AEs (incidence ≥15% and more than placebo) among the DM/Q recipients (*n = *76) were dizziness, nausea, and headache.

This patient population is susceptible to falls. Falls were reported by 16.5% of patients in this study; however, only six patients (1.1%) experienced a fall that was deemed by investigators as possibly related to treatment. In double-blind clinical trials falls were reported with similar incidence in DM/Q and placebo recipients.

Several types of AEs, particularly SAEs, were attributed to primary neurological conditions, for example respiratory depression, dyspnea, and dysphagia in ALS and disease aggravation in MS. Underlying disease contribution to AEs is supported by the AE analysis by disease group, AE data from DM/Q double-blind trials, and literature-reported mortality patterns in ALS patients^22–26^. Overall, the 39/176 ALS patient deaths constitute a mortality rate of 22.2%, which is lower than the death rate reported in prospective trials. In large-scale ALS-treatment trials, 1 year mortality rates ranged from approximately 26% to 35% in treatment arms and as high as 46% in placebo arms^27^,^28^. Furthermore, epidemiological studies generally suggest median survival after ALS diagnosis is approximately 15 to 19 months^29–34^, although at least one study has reported median survival post-diagnosis of 30 months^35^. The median survival after ALS diagnosis in the current study was estimated at 29 months (*n = *176); however, inferences regarding potential effect of DM/Q on survival are not possible without controlled studies.

The DM/Q 30/30 mg twice daily dosage administered in this study is greater than the DM/Q 20/10 mg twice daily dosage approved by the FDA and EMA for PBA treatment and also greater than the 30/10 mg twice daily dosage approved by the EMA. Given that the incidence of some TRAEs, such as dizziness and nausea, appear dose-related in controlled trials^22–24^, it is likely that the AE incidence reported in this long-term study would be greater than observed with the lower approved doses. Indeed, among patients treated with DM/Q 20/10 mg for 12 weeks during the pivotal trial (*n = *107) the percentages reporting headache, dizziness, and nausea were 14.0%, 10.3%, and 7.5%^24^ versus 22.8%, 19.5%, and 24.8%, respectively, in the present study.

Quinidine is a potent and reliable inhibitor of CYP2D6, and the dose in the approved DM/Q formulation (10 mg twice daily) is sufficient to increase DM exposure approximately 20-fold^24^. Other medications metabolized by CYP2D6 include antidepressants, antipsychotics, and beta-blockers. While concentrations of these medications may be increased in the presence of quinidine, potential drug interactions can be managed with simple dose adjustments. The overall number and type of concomitant medications taken in this study appear to reflect those seen in typical clinical practice. Patients who withdrew from the study for AEs did not appear to have a higher medication burden than those who completed the trial.

While quinidine is a well established type 1a antiarrhythmic drug with potential to cause dose-related QT-interval prolongation^36^, ventricular arrhythmia, and torsades de pointes^37–40^, no clinically significant arrhythmias were reported in controlled trials of DM/Q for PBA utilizing either the 30 or 10 mg doses of quinidine^22–24^. No patient had QTc increase >500 msec during the treatment phase. Two patients (both with MS) had a QTc >500 msec during the elective treatment extension; one had QTcB/QTcF = 493/505 msec and was withdrawn from the trial, and the other had prolonged QTc on two occasions (highest QTcB/QTcF = 526/506 msec on Day 1183) and remained in treatment until study termination. Overall ECG results suggested no clinically meaningful effect of DM/Q on myocardial repolarization or any ECG variable.

In summary, no significant ECG changes were observed on DM/Q during this 1 year study. The observed TRAEs occurred early in the treatment course, were largely mild-to-moderate, predominantly transient, and typically did not result in discontinuation. The safety and tolerability profile of DM/Q in this study was consistent with that observed in previous DM/Q trials, and the outcomes for patients receiving DM/Q followed the anticipated clinical course of the underlying neurological conditions.

## Key points

- Pseudobulbar Affect (PBA) is an uncontrollable disorder of emotional expression, occurring in a broad range of neurological diseases or injuries affecting the brain.
- Dextromethorphan/quinidine is the only medication approved by the US Food and Drug Administration and European Medicines Authority for the treatment of PBA.
- This 52 week open-label study of 553 patients demonstrates the long-term safety and tolerability of dextromethorphan and quinidine in patients with PBA secondary to a wide variety of neurological conditions.

## Supplementary Material

Supplementary Material

## Transparency

### Declaration of financial/other relationships

G.L.P. has disclosed that he has served on the speakers bureau for and received research support from Avanir Pharmaceuticals Inc. J.P.W. has disclosed that he has served on the speakers bureau for and received research support from Avanir Pharmaceuticals Inc. C.L.-H. has disclosed that she has no significant relationships with or financial interests in any commercial companies related to this study or article. S.H.A. has disclosed that he serves on a scientific advisory board for Neuraltus Pharmaceuticals Inc.; has received a speaker honorarium from Avanir Pharmaceuticals Inc.; receives research support from the National Institutes of Health and the Muscular Dystrophy Association; and has served as an expert consultant in a medicolegal case. A.E.F. and L.E.P. have disclosed that they are employees of Avanir Pharmaceuticals Inc.

The *CMRO* peer reviewer on this manuscript has received an honorarium from *CMRO* for review work, but has no relevant financial or other relationships to disclose.
