# Supplementary material for: An open-label multicenter study to assess the safety of dextromethorphan/quinidine in patients with pseudobulbar affect associated with a range of underlying neurological conditions
Source: Curr Med Res Opin. 2014 Jul 28;30(11):2255–65. doi: 10.1185/03007995.2014.940040 (PMC4743597; doi:10.1185/03007995.2014.940040)
Supplement: Supplementary Material [file icmo_a_940040_sm0001.pdf]

An Open-Label Multicenter Study to Assess the Safety of Dextromethorphan-Quinidine in Patients with Pseudobulbar Affect Associated with a Range of Underlying Neurological Conditions

Journal name: *CNS Drugs*

Gary L. Pattee, James P. Wymer, Catherine Lomen-Hoerth, Stanley H. Appel, Andrea E. Formella, and Laura E. Pope

Corresponding Author:
Gary L. Pattee, MD
Neurology Associates, PC
2631 South 70th Street
Lincoln, NE 68506, USA
e-mail: glpattee@gmail.com

Online Resource 1 Post hoc analysis of treatment-related adverse events (TRAEs)

TRAE[a]	Patients with TRAE (*n*)	Time to first onset of TRAE (median days)[b]	Duration of TRAE (median days)[c]	Patients with recurrent TRAE[d] (*n*)	Study days with TRAE present[e] (median %)
**All subjects: *N* = 553**					
Nausea	65	4.0	3.0	13	3.0%
Dizziness	58	4.5	2.0	12	2.0%
Headache	55	7.0	2.0	23	2.0%
Somnolence	39	8.0	8.0	10	21.5%
Fatigue	39	8.0	16.0	11	22.0%
Diarrhea	36	7.5	2.0	5	5.5%
Dry mouth	28	4.5	3.0	3	16.0%
**Patients who did not discontinue (for any reason) prior to TRAE resolution[f]**					
Nausea	49	—	2.0	9	1.0%

Dizziness	42	—	2.0	9	1.0%
Headache	46	—	2.0	22	1.0%
Somnolence	31	—	11.0	6	9.0%
Fatigue	32	—	21.5	9	11.5%
Diarrhea	26	—	2.0	5	1.5%
Dry mouth	20	—	5.5	1	8.0%
	Median time to discontinuation for any TRAE: 16.5 days				

[a] TRAEs are those where relationship to treatment is assessed as possible, probable, highly probable, or missing. Median onset and duration are computed only for patients with a TRAE

[b] Time in days from first dose to first onset of the TRAE during the treatment phase

[c] Duration in days from TRAE onset to TRAE resolution. For patients with more than one occurrence of the TRAE, the longest duration is reported. For subjects who discontinued prior to resolution, the date of last dose during the treatment phase is used to compute duration

[d] Number who experience the specific TRAE more than once

[e] Percentage of study days with TRAE present for each patient is the number of total study days with this TRAE (includes all occurrences of the AE) divided by total number of days patient participated in study. Total participation for each patient equals date of last dose minus date of first dose plus 1. If date of last dose is missing, date of last visit is used

[f] Excludes patients who discontinued for any reason prior to resolution of the TRAE in question. Analyzing only patients who did not discontinue prior to TRAE resolution avoids artificially censoring the TRAE duration due to dropout; onset is not calculated for this group since TRAE onset is not affected by subsequent discontinuation

An Open-Label Multicenter Study to Assess the Safety of Dextromethorphan-Quinidine in Patients with Pseudobulbar Affect Associated with a Range of Underlying Neurological Conditions

Journal name: *CNS Drugs*

Gary L. Pattee, James P. Wymer, Catherine Lomen-Hoerth, Stanley H. Appel, Andrea E. Formella, and Laura E. Pope

Corresponding Author:
Gary L. Pattee, MD
Neurology Associates, PC
2631 South 70th Street
Lincoln, NE 68506, USA
e-mail: glpattee@gmail.com

Online Resource 2 Incidence of SAEs reported during the study by ≥2 patients in any primary-neurological-condition category (safety population)

Primary neurological[a] condition	SAE	% (*n*)
AD/dementia	Cerebrovascular accident	11.8 (2)
MS	MS aggravated	5.8 (13)
	Myocardial infarction	0.9 (2)
	DVT	0.9 (2)
Stroke/CBVD	Cerebrovascular accident	3.9 (2)
ALS/MND	Respiratory failure	18.1 (36)
	Dysphagia	9.5 (19)
	Pneumonia NOS	4.0 (8)
	Amyotrophic lateral sclerosis	2.0 (4)
	Weight decreased	2.0 (4)
	Cardiac arrest	1.5 (3)
	Laceration	1.5 (3)
	Dyspnea	1.5 (3)
	Gastrostomy	1.5 (3)
	Hip fracture	1.5 (3)
	Epistaxis	1.0 (2)
	Aspiration pneumonia	1.0 (2)
	Postoperative wound infection	1.0 (2)

AD Alzheimer's disease, *ALS* amyotrophic lateral sclerosis, *CBVD* cerebrovascular disorders, *DVT* deep-vein thrombosis, *MND* motor neuron disease, *MS* multiple sclerosis, *NOS* not otherwise specified, *PBA* pseudobulbar affect, *PD* Parkinson's disease, *SAE* serious adverse event, *TBI* traumatic brain injury

[a] No SAE occurred in ≥2 patients in the TBI, PD/movement disorders, or other PBA groups

An Open-Label Multicenter Study to Assess the Safety of Dextromethorphan-Quinidine in Patients with Pseudobulbar Affect Associated with a Range of Underlying Neurological Conditions

Journal name: *CNS Drugs*

Gary L. Pattee, James P. Wymer, Catherine Lomen-Hoerth, Stanley H. Appel, Andrea E. Formella, and Laura E. Pope

Corresponding Author:
Gary L. Pattee, MD
Neurology Associates, PC
2631 South 70th Street
Lincoln, NE 68506, USA
e-mail: glpattee@gmail.com

Online Resource 3 AEs leading to discontinuation of ≥1% of patients (safety population)

**Preferred term, % (n)**	**AD/dementia (*n* = 17)**	**MS (*n* = 223)**	**Stroke/CBVD (*n* = 51)**	**TBI (*n* = 23)**	**ALS/MND (*n* = 199)**	**PD/movement disorders (*n* = 23)**	**Other PBA (*n* = 17)**	**Total (*N* = 553)**
Any AE	35.3 (6)	16.6 (37)	23.5 (12)	13.0 (3)	40.2 (80)	39.1 (9)	11.8 (2)	26.9 (149)
Respiratory failure	0 (0)	0 (0)	0 (0)	0 (0)	14.1 (28)	0 (0)	0 (0)	5.1 (28)
Nausea	0 (0)	1.8 (4)	2.0 (1)	0 (0)	6.0 (12)	4.3 (1)	0 (0)	3.3 (18)
Dizziness	0 (0)	2.2 (5)	3.9 (2)	0 (0)	2.0 (4)	17.4 (4)	5.9 (1)	2.9 (16)
Headache	0 (0)	1.8 (4)	2.0 (1)	0 (0)	2.5 (5)	8.7 (2)	5.9 (1)	2.4 (13)
Diarrhea	5.9 (1)	0.4 (1)	2.0 (1)	0 (0)	3.0 (6)	0 (0)	5.9 (1)	1.8 (10)

Somnolence	0 (0)	1.8 (4)	3.9 (2)	0 (0)	2.0 (4)	0 (0)	0 (0)	1.8 (10)
Weakness	0 (0)	0.9 (2)	2.0 (1)	4.3 (1)	2.0 (4)	4.3 (1)	0 (0)	1.6 (9)
Dry mouth	0 (0)	1.3 (3)	2.0 (1)	0 (0)	2.0 (4)	0 (0)	0 (0)	1.4 (8)
Fatigue	0 (0)	1.3 (3)	0 (0)	4.3 (1)	1.5 (3)	4.3 (1)	0 (0)	1.4 (8)
Insomnia	0 (0)	0.4 (1)	3.9 (2)	0 (0)	2.0 (4)	0 (0)	0 (0)	1.3 (7)
Appetite decreased	5.9 (1)	0.4 (1)	0 (0)	0 (0)	2.0 (4)	0 (0)	0 (0)	1.1 (6)

AD Alzheimer's disease, *AE* adverse event, *ALS* amyotrophic lateral sclerosis, *CBVD* cerebrovascular disorders, *MND* motor neuron disease, *MS* multiple sclerosis, *PBA* pseudobulbar affect, *PD* Parkinson's disease, *TBI* traumatic brain injury

An Open-Label Multicenter Study to Assess the Safety of Dextromethorphan-Quinidine in Patients with Pseudobulbar Affect Associated with a Range of Underlying Neurological Conditions

Journal name: *CNS Drugs*

Gary L. Pattee, James P. Wymer, Catherine Lomen-Hoerth, Stanley H. Appel, Andrea E. Formella, and Laura E. Pope

Corresponding Author:
Gary L. Pattee, MD
Neurology Associates, PC
2631 South 70th Street
Lincoln, NE 68506, USA
e-mail: glpattee@gmail.com

Online Resource 4 QT-interval changes

Parameter/ time point	Absolute value			Change from screening value		
	n	Statistic	Value	*n*	Statistic	Value
**QTcF, msec**						
Screening	550	Mean (SD)	402 (19.1)	n/a	n/a	n/a
		Median (min, max)	402 (345, 463)			
Day 29	465	Mean (SD)	405 (18.8)	463	Mean (SD)	+3.1 (15.9)
		Median (min, max)	405 (359, 476)		Median (min, max)	+2.2 (−76, 52)
Week 52/ Final Visit	399	Mean (SD)	406 (20.3)	396	Mean (SD)	+3.2 (18.3)
		Median (min, max)	405 (283, 480)		Median (min, max)	+3.4 (−125, 66)
**QTcB, msec**						
Screening	550	Mean (SD)	412 (21.3)	n/a	n/a	n/a
		Median (min, max)	410 (349, 479)			
Day 29	465	Mean (SD)	415 (20.0)	463	Mean (SD)	+3.7 (18.1)
		Median (min, max)	415 (365, 473)		Median (min, max)	+3.0 (−65, 69)
Week 52/ Final Visit	399	Mean (SD)	416 (21.7)	396	Mean (SD)	+4.6 (21.5)
		Median (min, max)	414 (294, 491)		Median (min, max)	+5.0 (−123, 83)

n/a not available, QTcB QT interval corrected by Bazett's method, *QTcF* QT interval corrected by Fredericia's method, *SD* standard deviation
